# A vision and a milestone: 10 years of pneumonia

**DOI:** 10.1186/s41479-022-00104-2

**Published:** 2023-01-11

**Authors:** Ger T Rijkers, Stephen I Pelton

**Affiliations:** 1 Science Department, University College Roosevelt, Middelburg, The Netherlands; 2 Department of Pediatrics, Section of Infectious Diseases, Boston University Chobanian & Avedisian School of Medicine, Boston, MA USA

## Pneumonia

In his famous textbook The Principles and Practice of Medicine, William Osler wrote in 1892 on pneumonia that it was a disease which carries off one in every four or five of those attacked. He further wrote that in children and in healthy adults the outlook is good, but in the debilitated, drunkards, and in the aged the chances are against recovery. While he probably was right on the burden of pneumonia for the debilitated, the drunkards, and the elderly, his positive tone for children was limited to a local perspective. Now, over a century later, and with antibiotics and vaccines available, as well as better housing and living conditions, still fourteen percent of all (global) childhood mortality is due to pneumonia. For the 740,000 children less than 5 years of age who succumbed to pneumonia in 2019 the outlook was not good. Prevention of pneumonias deaths thus still requires addressing nutrition, pollution, access to vaccines and early immunization, and access to effective antibiotics.

## Pneumonia floors

**Fig. 1:**
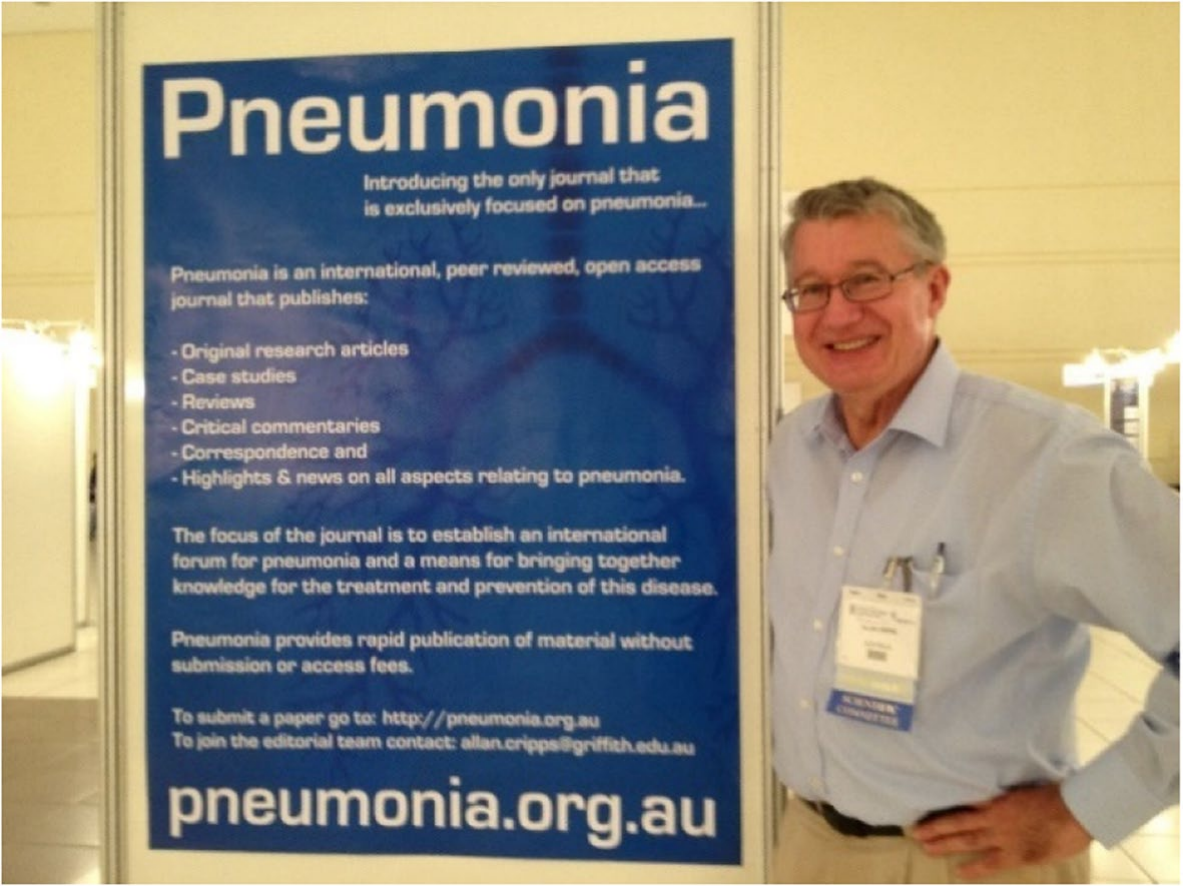
Founding editor of Pneumonia, Alan Cripps, announcing the birth of the journal on March 11, 2012

In 1966, John Lennon and Paul McCartney had an appointment with Bob Dylan, who at that moment was staying in the London Hilton. John and Paul wanted to learn from Bob Dylan how to be more creative in writing song lyrics. In a single session, under the influence of certain substances, the lyrics for a song were type-written on Hilton stationary. It doesn’t have a melody and was never performed. The title is pneumonia ceilings, pneumonia floors (Mark Shipper, Paperback Writer, Ace Publisher, London,1980). Pneumonia floors can be interpreted as the launch of the journal Pneumonia at the International Symposium on Pneumococci and Pneumococcal Diseases (ISPPD) at Foz de Iguacu in Brazil (Fig. 1) in 2012. It emerged from a vision shared by Alan Cripps and Bob Douglas and 43 delegates at a tri-national meeting (Indonesia, Papua New Guinea and Australia) in Sydney, Australia in 2009 on childhood pneumonia. Their vision was to establish an international forum for pneumonia for bringing together knowledge related to the pathogenesis, treatment and prevention of pneumonia.

Dr. Alan Cripps, Griffith University Health Pro Vice Chancellor, Australia, was the inaugural editor and chief for Pneumonia. His vision gave voice to research on pneumonia, especially the scientific community in emerging nations. The aspiration was to raise the global profile and awareness of pneumonia and advance the fight against childhood mortality. To this end, Pneumonia was launched and is an online, open access journal. Alan recruited a prestigious editorial board, passionate and dedicated to advancing the fight against childhood mortality due to pneumonia.

Volume 1, Issue 1 was published in December 2012. Early volumes of Pneumonia included publications from Malawi, Australia and France. The promise and threat to the success of pneumococcal conjugate vaccines, specifically serotype diversity and replacement and the financial challenge of global implementation were important themes. We now mark 10 years of activity growing from the initial visions of Dr. Allan Cripps and colleagues and brought to reality by the dedicated work of Allan, colleagues, board members, investigators, reviewers, staff, and the people from BioMed central.

The ten most accessed articles provide insight into the broad spectrum of publications and the areas of most interest to our readership (Table 1). Some of these introduced new concepts in pneumonia and have launched further studies in their themes. Themes such as long-term effects of pneumonia, duration of antimicrobial therapy in children, pulmonary impact of vaping, viral bacterial interactions remain at the forefront of challenges today in improving the outcome of pneumonia in both children and adults.

**Table 1:** Top 10 most downloaded manuscripts in Pneumonia in 2012-2021

Authors	Title	Year of publication	Downloads	Cited
Keith Grimwood and Anne Chang	Long-term effects of pneumonia in young children. https://link.springer.com/content/pdf/10.15172/pneu.2015.6/671.pdf	2015	88,616	39
Grant Mackenzie	The definition and classification of pneumonia. https://pneumonia.biomedcentral.com/articles/10.1186/s41479-016-0012-z	2016	79,759	138
Charles Feldman and Ronald Anderson	The role of co-infections and secondary infections in patients with COVID-19. https://link.springer.com/article/10.1186/s41479-021-00083-w	2021	62,812	143
Dan Wootton and Charles Feldman	The diagnosis of pneumonia requires a chest radiograph (x-ray)—yes, no or sometimes? https://pneumonia.biomedcentral.com/articles/10.15172/pneu.2014.5/464	2014	51,188	26
Brent Masters, Alan Isles, and Keith Grimwood	Necrotizing pneumonia: an emerging problem in children? https://link.springer.com/article/10.1186/s41479-017-0035-0	2017	49,991	96
Ashleigh Trimble, V Moffat, and Andrea Collins	Pulmonary infections in the returned traveler https://link.springer.com/article/10.1186/s41479-017-0026-1	2017	47,556	10
Davide Campagna, Maria Domenica Amaradio, Mark Sands, and Riccardo Pilosa.	Respiratory infections and pneumonia: potential benefits of switching from smoking to vaping. https://link.springer.com/article/10.1186/s41479-016-0001-2	2016	47,282	23
Kerry-Ann O’Grady, Paul Tornillo, Kieran Frawley, and Anne Chang	The radiological diagnosis of pneumonia in children. https://link.springer.com/content/pdf/10.15172/pneu.2014.5/482.pdf	2014	45,055	57
Katharina Yang, Robert Kruse, Weijjie Lin, and Dan Musher	Corynebacteria as a cause of pulmonary infection: a case series and literature review. https://link.springer.com/article/10.1186/s41479-018-0054-5	2018	28,500	37
Keith Grimwood, Siew Fong, Mong Ooi, Anna Nathan, and Anne Chang	Antibiotics in childhood pneumonia: how long is long enough? https://pneumonia.biomedcentral.com/articles/10.1186/s41479-016-0006-x	2016	21,783	15

## Pneumonia ceilings

In celebration of our 10-year milestone, we have commissioned manuscripts from esteemed scholars for Pneumonia to reflect on the current state of the art. We published the first of these, The Remarkable History of Pneumococcal Vaccination: An Ongoing Challenge, by Daniel Musher, Ronald Anderson and Charles Feldman. It has already had 1485 downloads (as of December 13, 2022) and provides an outstanding overview of the success and challenges in immunoprophylaxis against pneumococcal disease. Manuscripts on Viral bacterial interactions in pneumonia (Jason Rosch), Acute Organ Injury and Long-term sequelae of severe pneumococcal infection (Carlos J. Oriheula and Dawn Bowdish), History and prospects for pneumococcal vaccination in Malaysia (Alex Lister, David Cleary, and Stuart Clarke) and Tuberculosis: Prospects for eradication (Attapon Cheepsattayakorn) are in queue to be published in 2023. We believe these manuscripts will complement the original research published on line and enhance the value of Pneumonia to our readership.

The burden and toll of pneumonia continue to be substantial. We have made progress in the last decade with increased access to pneumococcal vaccination, expanded understanding of the linkage between pollution and respiratory health, viral bacterial interaction in the pathogenesis and the challenge of antimicrobial resistance, especially in nosocomial disease. There is more to be accomplished. We continue the early vision to present new and provocative science on Pneumonia with an emphasis on international investigators from both the paediatric and adult community. We continue to envision Pneumonia as a home for new insights into pneumonia in all of its aspects. The COVID-19 pandemic has taught us many lessons for the future. It certainly also has demonstrated that newly emerging infections can lead to severe pneumonia, that treatment of pneumonia remains to be a point of concern, and that sometimes existing drugs can be repurposed to control excessive pulmonary inflammation. The ceiling of pneumonia has not been reached.

With new hopes for 2023

Editors in Chief

Ger T Rijkers, PhD

Stephen I Pelton, MD

